# Relationship between hypoparathyroidism and the number of parathyroid glands preserved during thyroidectomy

**DOI:** 10.1186/1477-7819-12-200

**Published:** 2014-07-07

**Authors:** Chang Myeon Song, Joo Hwan Jung, Yong Bae Ji, Hyun Jung Min, You Hern Ahn, Kyung Tae

**Affiliations:** 1 Department of Otolaryngology-Head and Neck Surgery, College of Medicine, Hanyang University, 222 Wangsimniro, Seongdong-Gu, Seoul 133-792, South Korea; 2 Department of Internal Medicine, College of Medicine, Hanyang University, 222 Wangsimniro, Seongdong-Gu, Seoul 133-792, South Korea

**Keywords:** Thyroidectomy, Parathyroid, Thyroid cancer, Hypoparathyroidism, Hypocalcemia

## Abstract

**Background:**

The relationship between the number of parathyroid glands preserved and hypoparathyroidism is not well understood. We sought to determine the number of parathyroid glands that need to be preserved to prevent hypoparathyroidism.

**Methods:**

We analyzed 454 patients who underwent total thyroidectomy for papillary thyroid carcinoma. We analyzed the frequency of hypoparathyroidism according to the number of parathyroid glands preserved.

**Results:**

Incidental parathyroidectomy occurred in 19.8% of the patients; one parathyroid gland in 17.6%, two in 1.5%, and three in 0.7%. Transient hypoparathyroidism was increased when incidental parathyroidectomy occurred (odds ratio 1.83, 95% confidence interval 1.04 to 3.23, *P* = 0.036) on multivariate regression analysis, but was not influenced by the actual number of parathyroid glands removed. There was no relationship between the number of parathyroid glands preserved and permanent hypoparathyroidism (*P* = 0.147).

**Conclusions:**

Preservation of all parathyroid glands decreases transient hypoparathyroidism compared with when three or fewer glands are preserved, but does not affect permanent hypoparathyroidism. During total thyroidectomy, preserving at least one parathyroid gland with an intact blood supply appears to be sufficient to prevent permanent hypoparathyroidism when autotransplantation is not performed.

## Background

Hypoparathyroidism is a major complication of thyroidectomy. The incidence of transient hypoparathyroidism after thyroidectomy is reported to be about 10 to 46% while that of permanent hypoparathyroidism is as low as zero and as high as 43% [1-3]. Postoperative hypoparathyroidism increases the use of medication and biochemical tests, and prolongs hospital stay, so adding to the overall cost of thyroidectomy [4]. Preserving the parathyroid gland and its blood supply is the key to minimizing hypoparathyroidism following thyroidectomy. However, the relationship between the number of parathyroid glands preserved and hypoparathyroidism is not well understood. The aim of this study was to evaluate the clinical characteristics and the frequency of hypoparathyroidism according to the number of parathyroid glands preserved during thyroidectomy, and to determine the minimum number of parathyroid glands that need to be preserved to prevent hypoparathyroidism.

## Methods

### Patient selection and data collection

**Figure 1:**
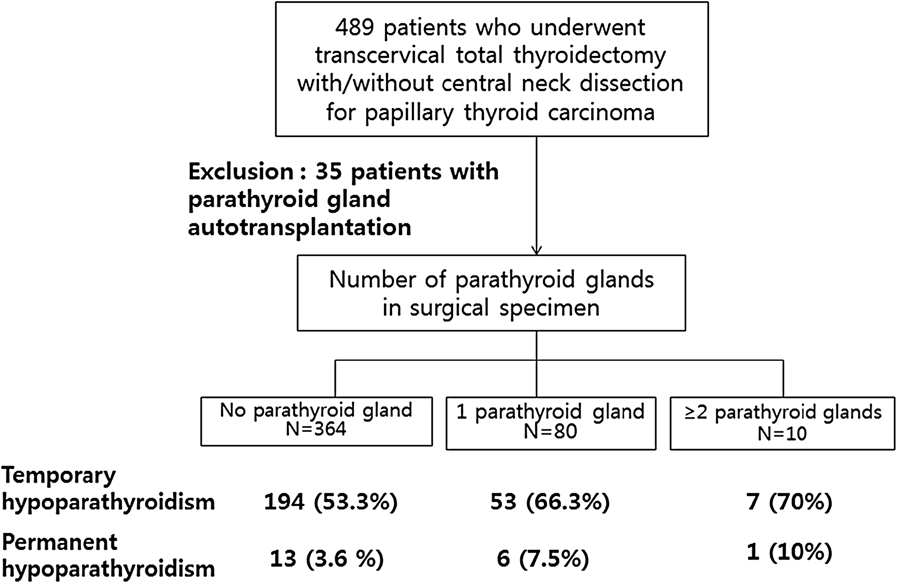
**The patient flow chart.** Thirty-five patients who underwent parathyroid autotransplantation were excluded. Patients were grouped according to the number of parathyroid glands found in the surgical specimen on histological examination, and the rate of hypoparathyroidism was compared between the groups.

A retrospective review was performed of 454 patients who underwent transcervical total thyroidectomy with or without neck dissection for papillary thyroid carcinoma between June 2007 and May 2011. We excluded 35 patients who had undergone parathyroid gland autotransplantation (Figure 1). Patients who had preoperative alterations of parathyroid function, severe chronic renal insufficiency, or diseases that interfered with calcium homeostasis, and who underwent robotic or endoscopic thyroidectomy, or completion or revision thyroidectomy were not included in the current study. The study was approved by the Institutional Review Board of Hanyang University Hospital.

We analyzed patient and tumor characteristics, extent of central and lateral neck dissection, number of parathyroid glands preserved, number found incidentally in the surgical specimens, the need for administration of intravenous calcium supplements, hypoparathyroidism, and other surgical complications. The operative procedure included capsular dissection and identification of the parathyroid glands to preserve the parathyroid glands and its vasculature during the central neck dissection (CND). The parathyroid glands were preserved with its blood supply from the inferior thyroid artery and its branches. We identified all superior parathyroid glands, and inferior parathyroid glands were identified in cases when CND was needed. In our institution, we do not routinely excise and transplant the parathyroid glands, especially in the dissection of inferior parathyroid glands. Only when devascularization of the parathyroid gland was evident, was autotransplantation to the ipsilateral sternocleidomastoid muscle performed after confirmation by frozen section. Therefore, we excluded cases with autotransplantation to reduce bias. Thyroidectomy was performed by two experienced thyroid surgeons (KT, YBJ). CND was performed according to the preference of surgeons, and bilateral CND was routinely performed by one surgeon (KT) when total thyroidectomy was indicated.

The number of parathyroid glands preserved was obtained by subtracting the number of parathyroid glands in a given specimen from four, because it is not always possible to identify all the parathyroid glands during thyroidectomy. Parathyroid function was measured as intact parathyroid hormone (iPTH), because iPTH has a short biological half-life and so is an appropriate index of parathyroid gland function [5-7]. Intact PTH was estimated by the electrochemiluminescence immunoassay (ECLIA) on Elecsys immunoassay analyzer (Roche, Indianapolis, IN, USA). Baseline levels of iPTH, total calcium (Ca), and ionized Ca were evaluated preoperatively, and their postoperative levels were measured after 24 hours after skin closure, 1 week, and 6 months, and when necessary. The decreased ratio of iPTH was calculated as:

(preoperativeiPTH‒postoperativeiPTH)×100/preoperativeiPTH

Hypoparathyroidism was defined as any drop in serum iPTH below the normal limit (normal range, 15 to 65 pg/mL), regardless of hypocalcemic symptoms. Permanent hypoparathyroidism was defined as lack of recovery of the iPTH to the normal range within six months [1,8-10]. Hypocalcemia was defined as decreased total calcium level (normal range, 8.4 to 10.4 mg/dl) or decreased ionized calcium level (normal range, 1.13 to 1.32 mmol/L) regardless of symptoms. All patients were followed up for at least six months after surgery.

The protocol for postoperative hypoparathyroidism is as follows: if patient showed hypocalcemia, oral calcium carbonate 500 mg three times a day and calcitriol 0.25 mcg daily were provided. If the medication was not sufficient, the dosage was increased to twice the dose. If hypocalcemic symptoms/signs including tingling sensation, Chvostek’s or Trousseau’s signs were present, intravenous 10% calcium gluconate 10 mL (94 mg elemental calcium) diluted with normal saline was administered.

### Statistical analysis

Data were analyzed using Statistical Package for the Social Sciences 18.0 for Windows (SPSS, Inc., Chicago, IL, USA). Fisher’s exact test was used to evaluate the differences of incidences and Mann-Whitney *U*-test was used to evaluate differences in means of variables between groups according to the parathyroid gland numbers excised. Multivariate analysis was performed to assess the independent risk factor for incidental parathyroidectomy and transient hypoparathyroidism. For multivariate analysis, unconditional logistic regression analysis was performed employing the variables that were statistically significant in univariate analysis. *P*-values less than 0.05 were considered statistically significant.

## Results

### Patients and tumor characteristics, surgical extents and outcomes

The patients consisted of 98 (22%) male and 356 (78%) females, ranging in age from 15 to 82 years, with a mean of 50.3 years. All the patients underwent total thyroidectomy, 122 patients (26.9%) without CND, 106 (23.3%) with unilateral CND, and 226 (49.8%) with bilateral CND. Seventy-one (15.6%) patients underwent lateral neck dissection together with CND.

Numbers of parathyroid glands detected in the surgical specimens by histological examination were as follows: 0 in 364 patients (80.2%), 1 in 80 patients (17.6%), 2 in 7 patients (1.5%), and 3 in 3 patients (0.7%). There were no examples of inadvertent excision of all four parathyroid glands. Various factors were compared according to the number of inadvertently excised parathyroid glands divided into 3 groups as follows: none (n = 364), 1 gland (n = 80), 2 or more glands (n = 10). Data on the patient and tumor characteristics associated with the number of excised parathyroid glands are given in Table 1. There was no association between the number of parathyroid glands excised and age, gender, tumor bilaterality, tumor location, or TNM stage. However, in univariate analysis, the group with one parathyroid excised had larger thyroid tumors, more frequent tumor bilaterality and minimal extrathyroidal extension, and more advanced T and N classification than the group with no incidental parathyroidectomy (*P* = 0.034, 0.030, 0.013, 0.045, and < 0.001, respectively). There was no significant difference in parameters other than N classification when we compared patients with one parathyroid gland excised and those with more than one excised (Table 1). N classification was higher in patients with two or more parathyroid glands removed than in those with only one gland removed (*P* = 0.046).

**Table 1:** Patient and tumor characteristics associated with the number of excised parathyroid glands

	**Number of parathyroid glands in resected specimen**				***P*-value**	
**Variable**	**0 (A) N = 364**	**1 (B) N = 80**	**2 or more (C) N = 10**	**Total n = 454**	**A versus B**	**B versus C**
Age	50.0 ± 12.5	52.2 ± 12.8	47.7 ± 6.08	50.3 ± 12.5	0.207	0.183
Gender					0.551	0.603
Male	81 (22.3%)	15 (18.7%)	2 (20%)	98 (21.6%)		
Female	283 (77.7%)	65 (81.3%)	8 (80%)	356 (78.4%)		
Tumor size	11.5 ± 9.3	12.5 ± 7.6	13.6 ± 10.8	11.7 ± 9.1	0.034	0.820
Tumor multiplicity	99/364 (27.2%)	32/80 (40%)	2/10 (20%)	133/454 (29.3%)	0.030	0.308
Tumor bilaterality	80/364 (22.0%)	23/80 (28.8%)	0	103/454 (22.7%)	0.192	0.059
Tumor location					0.212	0.150
Right	145 (39.8%)	23 (28.8%)	3 (30%)	171 (37.7%)		
Left	128 (35.2%)	32 (40%)	7 (70%)	167 (36.8%)		
Bilateral	77 (21.2%)	23 (28.8%)	0	100 (22.0%)		
Confined to isthmus	14 (3.8%)	2 (2.5%)	0	16 (3.5%)		
T classification					0.045	0.109
T1	203 (55.8%)	34 (42.5%)	4 (40.0%)	241 (53.1%)		
T2	24 (6.6%)	3 (3.8%)	1 (10.0%)	28 (6.2%)		
T3	134 (36.8%)	43 (53.8%)	4 (40.0%)	181 (39.9%)		
T4	3 (0.6%)	0	1 (10.0%)	4 (0.9%)		
N classification					< 0.001	0.046
N0	239 (65.7%)	35 (43.8%)	1 (10.0%)	275 (60.6%)		
N1	125 (34.3%)	45 (56.3%)	9 (90.0%)	179 (39.4%)		
TNM stage					0.090	0.769
I	226 (62.1%)	39 (48.8%)	4 (40.0%)	269 (59.3%)		
II	9 (2.5%)	1 (1.3%)	0	10 (2.2%)		
III	93 (25.5%)	27 (33.8%)	4 (40.0%)	124 (27.3%)		
IV	36 (9.9%)	13 (16.3%)	2 (20.0%)	51 (11.2%)		
Extrathyroidal extension (minimal)	157/364 (43.1%)	47/80 (58.8%)	5/10 (50%)	209/454 (46.0%)	0.013	0.737

We compared surgical extent, hypoparathyroidism, and other postoperative complications according to the number of parathyroid glands inadvertently excised (Table 2). Decreased ratio of iPTH showed a tendency to increase with increasing number of parathyroid glands excised, but these effects were not statistically significant. There were no differences in iPTH levels between patients with no inadvertent parathyroid glands and one parathyroid gland removed on day 1 after operation, or 1 week, and 6 months postoperatively. There were also no differences in permanent hypoparathyroidism, administration of intravenous calcium replacement, lateral neck dissection, operation time, vocal cord paralysis, or hematoma formation between the two groups. However, in univariate analysis, relative to patients with no parathyroid gland excised, those with one inadvertent parathyroid gland excised underwent more extensive CND, had more lymph nodes retrieved and also more metastatic lymph nodes in their central compartment, and more frequent temporary hypoparathyroidism (*P* < 0.001, < 0.001, 0.001, and 0.010, respectively). There were no differences in any measures when we compared patients with one parathyroid gland excised and those with more than one excised (Table 2).

**Table 2:** Surgical extent and complications associated with the number of excised parathyroid glands

	**Number of parathyroid glands in resected specimen**				***P*-value**	
**Variable**	**0 (A) N = 364**	**1(B) N = 80**	**2 or more (C) N = 10**	**Total n = 454**	**A versus B**	**B versus C**
CND					<0.001	0.749
None	118 (32.4%)	4 (5.0%)	0	122 (26.9%)		
Unilateral	91 (25.0%)	13 (16.3%)	2 (20.0%)	106 (23.3%)		
Bilateral	155 (42.6%)	63 (78.8%)	8 (80.0%)	226 (49.8%)		
Retrieved CLN (No)	5.0 ± 5.5	7.4 ± 5.2	7.7 ± 2.8	5.5 ± 5.5	< 0.001	0.641
Metastatic CLN (No)	1.2 ± 2.6	2.1 ± 3.3	2.3 ± 2.8	1.4 ± 2.8	0.001	0.748
Lateral ND	51/364 (14.0%)	18/80 (22.5%)	2/10 (20%)	71/454 (15.6%)	0.062	0.610
Operation time (minutes)	162.2 ± 73.7	179.1 ± 93.6	198.0 ± 142.5	166.0 ± 79.6	0.218	0.651
PTH, preop	38.35 ± 15.06	39.13 ± 14.09	34.23 ± 12.37	38.38 ± 14.81	0.701	0.432
PTH, postop 1 day	14.77 ± 11.82	11.71 ± 8.90	11.95 ± 11.32	14.15 ± 11.32	0.062	0.716
PTH, postop 1 week	21.39 ± 13.46	18.89 ± 11.12	17.07 ± 9.13	20.86 ± 13.02	0.125	0.813
PTH, postop 6 months	32.54 ± 15.04	31.25 ± 15.33	26.22 ± 14.87	32.11 ± 15.10	0.378	0.346
Dec. ratio, postop 1 day	58.65 ± 33.43	63.54 ± 35.55	67.55 ± 15.98	59.74 ± 33.49	0177	0.722
Dec. ratio, postop 1 week	40.52 ± 38.23	49.45 ± 24.49	41.39 ± 38	41.39 ± 38.63	0.248	0.910
Dec. ratio, postop 6 months	7.09 ± 53.72	13.24 ± 50.51	21.49 ± 43.53	8.71 ± 52.80	0.124	0.813
Hypoparathyroidism						
Temporary	194/364 (53.3%)	53/80 (66.3%)	7/10 (70%)	254 (55.9%)	0.010	0.697
Permanent	13/364 (3.6%)	6/80 (7.5%)	1/10 (10%)	20 (4.4%)	0.128	0.575
IV calcium administration	27/364 (7.4%)	10/80 (12.5%)	3/10 (30%)	40/454 (8.8%)	0.177	0.155
Vocal cord paralysis					0.787	0.093
None	351 (96.2%)	77 (96.3%)	8 (80%)	436 (96.0%)		
Temporary	12 (3.3%)	3 (3.8%)	2 (20%)	17 (3.7%)		
Permanent	1 (0.3%)	0	0	1 (0.2%)		
Hematoma	15/364 (4.1%)	4/80 (5%)	0	19/454 (4.2%)	0.760	0.619

CLN, central lymph node; CND, central neck dissection; Dec, decreased; IV, intravenous; ND, neck dissection; No, number; postop, postoperative; preop, preoperative; PTH, parathyroid hormone; d, day; w, week; m, month; postop, postoperative; preop, preoperative.

In multivariate logistic regression analysis, the extent of CND was the only independent risk factor for incidental parathyroidectomy (Table 3). The odds ratios of unilateral and bilateral CND for incidental parathyroidectomy were 3.7 and 9.3, respectively.

**Table 3:** Multivariate logistic regression for incidental parathyroidectomy

**Variable**	**β Coef**	**SE**	***P*-value**	**OR**	**95% (lower)**	**95% (upper)**
Tumor size	-0.016	0.016	0.337	0.985	0.954	1.016
Tumor multiplicity	0.232	0.282	0.411	1.261	0.726	2.190
T classification (advanced)	0.367	0.400	0.359	1.443	0.659	3.159
N classification	0.204	0.339	0.546	1.227	0.631	2.384
Minimal ETE	0.057	0.408	0.888	1.059	0.476	2.356
Excised central LN (No)	-0.004	0.030	0.890	0.996	0.939	1.056
Metastatic central LN (No)	0.011	0.054	0.845	1.011	0.909	1.123
CND			0.000			
Unilateral	1.306	0.611	0.033	3.691	1.115	12.222
Bilateral	2.228	0.585	0.000	9.280	2.950	29.198
Hypoparathyroidism			0.077			
Transient	0.556	0.296	0.061	1.743	0.976	3.116
Permanent	1.083	0.597	0.070	2.952	0.917	9.507

Coef, coefficient; OR, odds ratio; 95% lower, 95% confidence interval (lower); 95% upper, 95% confidence interval (upper); CND, central neck dissection; ETE, extrathyroidal extension; LN, lymph node; No, number.

### Factors affecting hypoparathyroidism

Transient and permanent hypoparathyroidism occurred in 254 (55.9%) and 20 patients (4.4%), respectively. On univariate analysis, the frequency of transient hypoparathyroidism rose when parathyroid glands were found in specimens, CND had been performed, or numbers of retrieved and metastatic central lymph nodes were higher (*P* = 0.004, 0.031, 0.007, and 0.016, respectively) (Table 4). The incidence of transient hypoparathyroidism also increased when there were 3 or fewer preserved parathyroid glands than when all 4 parathyroid glands were preserved (*P* = 0.004). However, among patients in whom one or more parathyroid gland was not preserved, the incidence of transient hypoparathyroidism did not increase with the number of parathyroid glands removed (preserved parathyroid glands 1 versus 2, 2 versus 3, *P* = 0.583 and 0.470, respectively). Patient and tumor characteristics, TNM stage, extrathyroidal extension, and complication of surgery did not differ between patients with and without transient hypoparathyroidism. In multivariate logistic regression analysis, only incidental parathyroid gland identified in a specimen was an independent risk factor for transient hypoparathyroidism (OR 1.83, 95% CI 1.04 to 3.23, *P* = 0.036). Pathologic N positive tumor was the sole risk factor for permanent hypoparathyroidism on multivariate analysis (Table 5). There was no relationship between the number of preserved parathyroid glands and permanent hypoparathyroidism.

**Table 4:** Univariate analysis of transient hypoparathyroidism

**Factors**	**Transient hypoparathyroidism**		***P*-value**
	**No n = 180**	**Yes n = 254**	
Age	51.8 ± 12.4	49.6 ± 12.5	0.075
Gender			0.411
Male	43 (23.9%)	52 (20.5%)	
Female	137 (76.1%)	202 (79.5%)	
Tumor size (mm)	12.1 ± 9.7	11.4 ± 8.6	0.398
Tumor location			0.899
Right	69 (38.3%)	97 (38.2%)	
Left	64 (35.6%)	97 (38.2%)	
Bilateral	41 (22.8%)	51 (20.1%)	
Confined to isthmus	6 (3.3%)	9 (3.5%)	
Tumor multiplicity	55/180 (3.6%)	72/254 (28.3%)	0.669
Tumor bilaterality	44/180 (24.4%)	52/254 (20.5%)	0.349
Preserved PTG number			0.025
4	157 (87.2%)	194 (76.4%)	
3	21 (11.75)	53 (20.9%)	0.010
2	1 (0.6%)	5 (2.0%)	0.470
1	1 (0.6%)	2 (0.8%)	0.583
Incidental parathyroidectomy	23/180 (12.8%)	60/254 (23.6%)	0.004
T classification			0.123
T1	98(54.4%)	136(53.5%)	
T2	15(8.3%)	11(4.3%)	
T3	64(35.6%)	106(41.7%)	
T4	3(1.7%)	1(0.4%)	
N classification			0.422
N0	116(64.4%)	153(60.2%)	
N1	64(35.6%)	101(39.8%)	
TNM stage			0.212
I	100(55.6%)	158(62.2%)	
II	7(3.9%)	3(1.2%)	
III	52(28.9%)	65(25.6%)	
IV	21(11.7%)	28(11.0%)	
Extrathyroidal extension (minimal)	79/180 (43.9%)	118/254 (46.5%)	0.625
CND (bilateral versus unilateral)	79/122(64.8%)	137/194(70.6%)	0.320
CND (yes versus no)	122/180 (67.8%)	194/254 (76.4%)	0.031
Lateral ND	26/180 (14.4%)	39/254 (15.4%)	0.892
Lateral LN metastasis			0.275
None	161 (89.4%)	218 (85.8%)	
Unilateral	13 (7.2%)	30 (11.8%)	
Bilateral	6 (3.3%)	6 (2.4%)	
Operation time (minutes)	160 ± 74	170 ± 84	0.215
Retrieved central LN (No)	4.6 ± 5.1	6.0 ± 5.6	0.007
Metastatic central LN (No)	1.0 ± 2.0	1.6 ± 3.2	0.016
Amount of drainage (mL)	224 ± 787	225 ± 619	0.985
Vocal cord paralysis			0.679
None	172(95.6%)	244(96.1%)	
Temporary	7(3.9%)	10(3.9%)	
Permanent	1(0.6%)	0	
Hematoma	8/180(4.4%)	11/254(4.3%)	0.567

CND, central neck dissection; LN, lymph node; ND, neck dissection; No, number; PTG, parathyroid gland.

**Table 5:** Univariate analysis of permanent hypoparathyroidism

	**Permanent hypoparathyroidism**		***P*-value**
**Factors**	**No n = 434**	**Yes n = 20**	
Age	50.5 ± 12.5	46.3 ± 12.2	0.059
Gender			0.587
Male	95 (21.9%)	3 (15.0%)	
Female	339(78.1%)	17 (85.0%)	
Tumor size (mm)	11.7 ± 9.1	12.9 ± 8.9	0.367
Tumor location			0.180
Right	166 (38.2%)	5 (25.0%)	
Left	161 (37.1%)	6 (30.0%)	
Bilateral	92 (21.2%)	8 (40.0%)	
Confined to isthmus	15 (3.5%)	1 (5.0%)	
Tumor multiplicity	127/434 (29.3%)	6/20 (30.0%)	0.559
Tumor bilaterality	96/434 (22.1%)	7/20 (35.0%)	0.179
Preserved PTG number			0.147
4	351 (80.9%)	13 (65.0%)	
3	74 (17.1%)	6 (30.0%)	
2	6 (1.4%)	1 (5.0%)	
1	3 (0.7%)	0	
Incidental parathyroidectomy	83/434 (19.1%)	7/20 (35.0%)	0.090
T classification			0.285
T1	234(53.9%)	7(35.0%)	
T2	26(6.0%)	2(10.0%)	
T3	170(39.2%)	11(55.0%)	
T4	4(0.9%)	0	
N classification			0.008
N0	269(62.0%)	6(30.0%)	
N1	165(38.0%)	14(70.0%)	
TNM staging			0.871
I	258 (59.4%)	11(55.0%)	
II	10 (2.3%)	0	
III	117 (27.0%)	7(35.0%)	
IV	49 (11.3%)	2(10.0%)	
Extrathyroidal extension (minimal)	197/434(45.4%)	12/20(60.0%)	0.253
CND			0.663
None	118 (27.2%)	4 (20.0%)	
Unilateral	100 (23.0%)	6 (30.0%)	
Bilateral	216 (49.8%)	10 (50.0%)	
Lateral ND	65/434 (15.0%)	6/20 (30.0%)	0.106
Lateral LN metastasis			0.683
None	379 (87.3%)	17 (85.0%)	
Unilateral	43 (9.9%)	3 (15.0%)	
Bilateral	12 (2.8%)	0	
Operation time (minutes)	186.1 ± 80.0	183.5 ± 74.5	0.841
Retrieved central LN (no)	5.5 ± 5.4	6.2 ± 6.6	0.743
Metastatic central LN (no)	1.4 ± 2.8	1.5 ± 2.4	0.355
Drain amount (mL)	224.7 ± 692.8	151.8 ± 152.4	0.188
Vocal cord paralysis			1.000
None	416(95.9%)	20(100%)	
Temporary	17(3.9%)	0	
Permanent	1(0.2%)	0	
Hematoma	19/434(4.4%)	0	1.000

CND, central neck dissection; LN, lymph node; ND, neck dissection; No, number; PTG, parathyroid gland.

## Discussion

To avoid hypoparathyroidism, it is important to preserve the parathyroid glands and their vascular supply during thyroid surgery. Despite meticulous care, parathyroid glands are occasionally found in the surgical specimens. Usually the parathyroid glands are located extracapsularly on the posterior surface of the thyroid gland. However, their location can vary; in one study they were found in extracapsular (58%), intracapsular (20%), and intrathyroidal (22%) locations [11]. The superior parathyroid glands are usually located at the superior pole of the posterior thyroid gland near the cricothyroid junction, while the inferior parathyroid glands are usually found in the lower pole of the thyroid gland, but can be located elsewhere including the thymus and mediastinum. Thus, it is not always possible to identify all four parathyroid glands. In an autopsy series of 503 cases, 17% of the inferior parathyroid glands were found on or within the capsule of the thyroid gland, whereas 26% were found within the cervical part of the thymus [12]. In another study, the parathyroid glands in the resected specimens were found to be intrathyroidal in 50% of the thyroidectomies [13]. Thus, it is more reliable to evaluate the number of preserved parathyroid glands from the number of parathyroid glands identified in the surgical specimens than from the number of preserved parathyroid glands seen intraoperatively by the surgeons with the naked eye. In this study, we estimated the number of preserved parathyroid glands by subtracting from four the number of parathyroid glands in the surgical specimen. However, a limitation of this method is that some patients have supernumerary parathyroid glands. In a cadaveric dissection of 942 patients, a fifth parathyroid gland was found in 5% of the individuals, and three parathyroid glands (instead of four) were found in 2% [14].

In the current study, tumor size, multiplicity, T classification, N classification, minimal extrathyroidal extension, extent of CND, numbers of retrieved and metastatic lymph nodes in the central compartment, and transient hypoparathyroidism were related to the presence of inadvertent parathyroid glands in the surgical specimen; however, only the extent of CND was an independent risk factor in multivariate analysis, with odd ratios of unilateral and bilateral CND of 3.7 and 9.3, respectively. Hence, surgeons should take care not to resect any of the parathyroid glands during CND, and especially, to preserve the inferior parathyroid gland. The incidence of inadvertently excised parathyroid gland in the current study was 19.8%, which is in accord with previous studies that had rates of 5 to 21% [11,13,15-17].

While identification of all four parathyroid glands is traditionally recommended to reduce postoperative hypoparathyroidism [15,18], there are recent studies suggesting that identification of a greater number of identified parathyroid glands intraoperatively does not reduce the incidence of hypoparathyroidism [19,20]. However, in cases with CND, we recommend identifying the parathyroid glands to perform a thorough CND, and to consider that CND without identifying the parathyroid gland can lead to insufficient lymph node dissection or blind trauma of parathyroid gland vasculature regardless of capsular dissection. Only a few studies have examined whether the number of parathyroid glands preserved affects the likelihood of hypoparathyroidism. In one study, the rate of permanent hypoparathyroidism increased if fewer than three parathyroid glands had been identified and preserved intraoperatively [21]. In another study, multivariate analysis in 5,846 patients showed that intraoperative identification and preservation of fewer than 2 parathyroid glands resulted in an increased rate of permanent postoperative hypoparathyroidism. No added benefit was found in elevating the number of identified parathyroid glands from two to three [22]. The current study differed from these previous studies, which evaluated numbers of preserved parathyroid glands intraoperatively, in that the number of parathyroid glands preserved was deduced indirectly from histopathological examination of the resected surgical specimens. The lack of histologic confirmation may result in overestimating rates of parathyroid gland conservation. Evaluating preserved parathyroid glands intraoperatively has drawbacks since the surgeon cannot always see all the parathyroid glands, and frozen biopsy is not always accurate.

Many previous studies of hypoparathyroidism included cases with heterogeneous features including different extents of thyroidectomy, and various types of thyroid lesion [4,23,24]. In the current study, to eliminate these confounding factors, we limited ourselves to patients who underwent total thyroidectomy, and the pathology was confined to papillary thyroid carcinoma. The incidence of transient hypoparathyroidism increased when one or more parathyroid gland was present in the surgical specimens (odd ratio 1.8), but it did not increase further as the number of excised glands increased. In one study evaluating the risk of hypoparathyroidism, the only independent variable related to transient hypoparathyroidism in multivariate analysis was the extent of surgery, not only of paratracheal groove dissection, but also of lateral neck dissection [4].

The incidence of permanent hypoparathyroidism in our study was 4.4%, which is in accordance with recent reports (1.9 to 7.1%), despite the high rate of CND performed in our study (73.1%) [19,25]. In a retrospective study of 1,087 patients who underwent total thyroidectomy for PTC, the rates for permanent hypoparathyroidism were 6.3% for patients without CND, 7% for unilateral CND, and 16.2% for bilateral CND [26].

One study compared the usefulness of postoperative iPTH and calcium in predicting permanent hypoparathyroidism [25]. Intact PTH levels measured at 24 hours after total thyroidectomy with a cut-off value of 5.8 pg/mL, ruled out permanent hypoparathyroidism more accurately (sensitivity 100%; specificity, 81.5%) than calcium levels (cut-off value 1.95 mmol/L, sensitivity, 60%; specificity, 78.5%) and recommended postoperative iPTH for evaluating the risk for developing permanent hypoparathyroidism [25]. A study comprising 27 published articles evaluating the value of iPTH revealed that iPTH was efficient to stratify the risk for developing hypocalcemia after thyroid surgery [27]. Our data are in accordance with the results. When we evaluated the diagnostic value of iPTH by a receiver operating characteristic (ROC) curve in our data, an iPTH value ≤ 9.8 pg/mL at 24 hours after surgery identified patients at risk for permanent hypoparathyroidism (sensitivity, 100%; specificity, 57.64%), but could not predict its development (positive predictive value, 9.8%). Conversely, an iPTH level > 9.8 pg/mL predicted normal parathyroid function at 6 months after thyroidectomy (negative predictive value, 100%). Also, an iPTH value > 5.9 pg/mL at postoperative 24 hours predicted hypocalcemic symptoms/sign as less likely to occur (negative predictive value, 97.88%). In our study, hypoparathyroidism, hypocalcemia, and hypocalcemic symptoms were all correlated with each other with statistical significance.

In this study, the number of preserved parathyroid glands did not affect the risk of permanent hypoparathyroidism, and positive N stage was the sole factor that increased this risk. There was no permanent hypoparathyroidism in any of the examples in this study of inadvertent excision of three parathyroid glands. Hence, our data suggest that the preservation of at least one viable parathyroid gland prevents permanent hypoparathyroidism. In addition to preserving as many viable parathyroid glands as possible, the blood supply of parathyroid gland should be preserved with great care and careful dissection of tissue to maintain the viability of the parathyroid glands.

The current study has limitations in that it is retrospective and without randomization of surgical extent, and the viability and vascularization of the preserved parathyroid glands was not confirmed. A further study involving the evaluation of the viability of the preserved parathyroid glands and the comparison with cases of parathyroid autotransplantation is needed.

## Conclusions

In conclusion, the preservation of all four parathyroid glands during thyroidectomy decreases the incidence of transient hypoparathyroidism, but is not required to prevent permanent hypoparathyroidism. It is likely that preserving at least one parathyroid gland with an intact blood supply is sufficient to prevent permanent hypoparathyroidism when autotransplantation is not performed.

### Consent

Written informed consent was obtained from the patient for the publication.

## Abbreviations

Ca: calcium; CND: central neck dissection; ECLIA: electrochemiluminescence immunoassay; iPTH: intact parathyroid hormone; OR: odds ratio; ROC: receiver operating characteristic.

## Competing interests

The authors declare that they have no competing interests or financial ties to disclose.

## Authors’ contribution

CMS and KT participated in the study design. CMS, JHJ, and YBJ participated in acquisition of data. HJM and YHA participated in interpretation of data and helped draft of manuscript. CMS and JHJ performed the literature review and data analysis. CMS and YBJ drafted the manuscript. YHA and KT revised the manuscript. All authors read and approved the final manuscript.
